# Drug policies’ sensitivity towards women, pregnancy, and motherhood: a content analysis of national policy and programs from nine countries and their adherence to international guidelines

**DOI:** 10.1186/s13722-023-00410-0

**Published:** 2023-09-08

**Authors:** Abhishek Ghosh, Dijana Jerkovic, Liljana Aleksandar Ignjatova, Carla Bruguera, Dalia I. Ibrahim, Katarzyna Okulicz-Kozaryn, J. Maphisa Maphisa, Thomas F. Martinelli, Ana Neto, Joana Canedo, Rebecca Gordon

**Affiliations:** 1 Department of Psychiatry, Postgraduate Institute of Medical Education & Research, Chandigarh, India; 2 Department of Criminology, Faculty of Education and Rehabilitation Sciences, University of Zagreb, Zagreb, Croatia; 3 Faculty of Medicine Skopje, University of St. Cyril and Methodius Skopje, Skopje, Republic of North Macedonia; 4 Program on Substance Abuse, Public Health Agency of Catalonia, Barcelona, Spain; 5 Mental Health and Addiction Treatment-Ministry of Health and Population-Egypt, Cairo, Egypt; 6 Institute of Mother and Child, Warsaw, Poland; 7 Department of Psychology, University of Botswana, Gaborone, Botswana; 8 Program Drugs, Trimbos-Institute, Utrecht, The Netherlands; 9 Unidade de Alcoologia de Lisboa, DICAD, ARSLVT, IP, Lisbon, Portugal; 10 Development Studies (ICS-IUL, FCT), EuroNPUD, GAT Manas, Lisbon, Portugal; 11 Addictions Unit, Psychiatry Department, Institute of Neurosciences, Hospital Clínic de Barcelona, Barcelona, Spain

## Abstract

**Background and objectives:**

Substance use in women is associated with unique psycho-social and physical vulnerabilities and poses complex challenges during pregnancy and motherhood. Gender-sensitive drug policy which considers the needs of women and their children could address these concerns. The objectives of this study were: (1) to systematically explore national-level drug policies’ sensitivity and responsiveness to women, pregnant women, and children; and (2) to examine the adherence of drug policies with international guidelines for gender sensitivity in drug policy.

**Methods:**

The research team was diverse professional backgrounds and nine countries. A summative content analysis of national drug policy documents, action plans, and strategies was performed. Specific documents focusing on women, pregnancy, and children were analysed. Specific themes and how frequently they appeared in the documents were identified. This quantification was an attempt to explore usage indicating the relative focus of the policies. A thematic map was developed to understand how national-level drug policies conceive and address specific concerns related to women who use drugs. We adapted the UNODC checklist for gender mainstreaming to assess policies’ adherence to international guidelines.

**Results:**

Twenty published documents from nine countries were reviewed. The common themes that emerged for women, pregnancy, and children were needs assessment, prevention, treatment, training, supply reduction, and collaboration and coordination. Custody of children was a unique theme for pregnant women. Specific psycho-social concerns and social reintegration were special themes for women, whereas legislation, harm reduction, research, and resource allocation were children-specific additional themes. For women-specific content analysis, special issues/concerns in women with drug misuse, need assessment, and prevention were the three most frequent themes; for the children-specific policies, prevention, training, and treatment comprised the three most occurring themes. For pregnant women/pregnancy, prevention, treatment, and child custody were the highest occurring themes. According to ratings of the countries’ policies, there is limited adherence to international guidelines which ensure activities are in sync with the specific needs of women, pregnant women and their children.

**Conclusion:**

Our analysis should help policymakers revise, update and adapt national policies to ensure they are gender-responsive and address the needs of women, pregnant women and their children.

## Background

Women comprise one-third of people who use drugs globally and account for one-fifth of the estimated global number of people who inject drugs [1]. Women comprised one-third of overdose deaths in the US and about one in four in Canada in 2017–18. The rate of fatal overdoses among women has increased by 260–500% in the last two decades [2]. Women also suffer serious long-term social and health consequences of incarceration related to drug use and drug-related offenses which are different to those suffered by men [3–5]. The latest European report on *Women and Drugs* estimated one in four people with serious drug problems and one in five entrants to treatment programs were women. Despite the disease burden, the report lamented limited availability of integrated and coordinated national-level gender-specific services and gender-mainstreaming responses of drug use-related problems [6].

Women face particular challenges related to drugs including gender, effects of drug use during pregnancy (e.g., neonatal abstinence syndrome, low birth weight, and premature birth), motherhood, gender-based violence, higher involvement in sex work, higher prevalence of (sexual) trauma, double stigma (being discriminated against for being a woman and persons who use drugs) with serious psychosocial consequences [7]. These challenges require gender-specific policy responses. Drug policy should be well-aligned with the objectives of sustainable development goals (SDG-2030), which envisage gender equality and empowerment [8]. Therefore, it is important to assess whether women who use drugs currently receive attention in drug policies and programs and in what ways. Assessing gender-specific elements of national drug policies may help to address the particular challenges faced by women who use drugs and improve access to rights- and evidence-based harm reduction, treatment, rehabilitation, and social reintegration programs adapted to the needs of women. Treatment services often are less accessible and do not align with the needs of women with substance use disorders, as they are designed to respond to the needs of the majority in treatment, namely men. Women who use drugs are typically stigmatized for their drug-use, marginalizing them from mainstream society. This can cause fear of legal sanctions and loss of child custody making them less likely to seek treatment. Women report social stigma in private and professional contexts as a barrier to seeking and accessing treatment [9].

Even though for most drug use remains lower among women than men, there are substances where these differences are less evident. For instance, the prevalence of non-medical use of tranquilizers and opioids remains comparable to that of men, if not higher in women [5, 10]. Underlying reasons for substance use may also differ, for example, self-medication after experiencing childhood adversity events and internalized behaviours. Women also tend to progress more rapidly than men from initiating substance use to developing substance use disorders known as telescoping [11]. Women who inject drugs have a higher risk for drug-related infectious diseases than men since they are likely to share injecting equipment with more people, and/or to trade sex for drugs or money while at the same time having difficulties negotiating condom use with sexual partners [12, 13].

Identifying and understanding gender differences in drug use and disorders is a starting point. It can help shape gender-sensitive policies and practices that respond to the needs of women and mothers who use drugs. A further issue which has a significant effect on women who use drugs and their children is policy and interventions which punish rather than support these women [12, 14–16]. The stigma and fear associated with loss of child custody and/or punitive legal measures often deter women from seeking treatment. Of particular concern is quasi-compulsory treatment delivered through child-protection services, practiced in several countries which lacks adequate scientific evidence [17].

Although gender in drug policies was first discussed at the 1984 Ministerial Conference of the Pompidou Group, the pioneer in Europe regarding the integration of the gender dimension into drug policies [10], in recent decades there has been a growing awareness of the importance of incorporating gender perspectives into national and international drug policies and practices. This has resulted in numerous publications and documents on drug policies and national and international drug strategies that give an increasingly significant place to gender perspectives.

The latest publications of the Pompidou Group related to the gender perspective in drug policy put gender sensitivity in drug responses into the focus of policymakers and practitioners, with an emphasis on integrating specific gender needs when providing services, as well as protecting the rights of children in families affected by substance use [7, 18].

The UNODC and WHO developed international standards for treating drug use disorders that will benefit policymakers, managers of health and social services, and practitioners working with people with drug use disorders. The standards aim to help achieve health target 3.5 of the United Nations 2030 Agenda for Sustainable Development by “strengthening prevention and treatment of substance abuse” and universal health coverage for people with drug use disorders. The standards will also support the evaluation and ongoing improvement of services and the development of new policies and treatment systems. Some of these quality standards are related to the fact that treatment services should be gender-sensitive and oriented toward the needs of the populations they serve. Services for women, pregnant women and women with children who use drugs should be non-discriminatory and comprehensive, and tailored to their needs. This is relevant for all aspects of intervention design and delivery, including location, staffing, program development, child-friendliness, and content [6, 19]. Maintaining or improving relationships with children may play a central role in women’s drug use and recovery [6]. Limited availability of evidence-based recommendations for identifying and managing substance use and substance use disorders in pregnancy, for managing neonatal abstinence syndrome, and care for children of parents who use drugs places women and their children at further risk of harm [20]. Therefore, we felt an urgent need to do an in-depth analysis of the drug policies’ sensitivity towards women, pregnancy, and motherhood.

This study aims to analyze nine countries’ national policies and programs from the perspectives of their sensitivity towards women, pregnancy, and motherhood. The two broad objectives were: (1) to systematically explore national-level drug policies/strategies/action plans’ sensitivity and responsiveness to women, pregnancy, and children; and (2) to examine the adherence of drug policies/strategies/action plans with international guidelines for gender sensitivity in drug policy. Our predominant focus is on illegal drugs. The countries were chosen according to the authors’ country of origin. We believe the authors’ familiarity of the content and language would allow an in-depth analysis of the national policies.

## Methods

### Research team

The research team was put together by the Intercontinental Perspectives on Global Addictions and Drug Markets (Inter-GLAM) project. Inter-GLAM is a European project, co-funded by the European Union’s DG Justice Programme “Drugs Policy Initiatives—Supporting initiatives in the field of drugs policy” (JUST-2019-AG-DRUGS) from July 2021 to end of June 2023. The mandate of the research team was to work on recognizing global diversity in public health responses.

The team comprised nine researchers, clinicians, and academics from diverse professional backgrounds. Three were addiction psychiatrists, two were drug policy experts, and there was one each from medicine, public health, psychology, social pedagogy, and social anthropology. The members were affiliated with public healthcare sectors (n = 3), academic institutions (n = 3), and research bodies (n = 3). The common denominator was their expertise in the public health concerns associated with drug and alcohol use and drug policy. The team members were from the European (n = 5), Eastern Mediterranean (n = 2), Asian (n = 1), and African (n = 1) regions. Each member contributed to collecting, organizing, and reviewing drug policy documents of their country of work.

### Study design

A summative content analysis was performed of the published national drug policy documents/action plans/strategies. A quantitative description of the frequencies of the identified themes was presented.

### Theoretical framework

The content analysis was not theory-driven, but it was data-driven. Preconceived coding categories were not used—codes and names for codes were allowed to emerge from the data. Researchers immersed themselves in the data to form new insights through the codes.

### Sampling and sample size

Twenty published national drug policies and related documents from nine countries (Botswana, Croatia, Egypt, India, North Macedonia, Netherlands, Poland, Portugal, and Spain) were reviewed.

### Search strategy

The authors provided the drug policy documents for their respective countries of origin. We included policy documents related to supply, demand, and harm reduction, published in any language and any year of publication. We included any revised policy documents. We excluded documents exclusively on legal substances (e.g., alcohol and tobacco); however, if any country had a common policy document for all substances, these were included. Moreover, for countries that did not have a national-level drug policy (e.g., Botswana), we included alcohol and tobacco policy reports and national health/public health policies.

### Method of approach

To search the relevant text matters within the policy documents, the following keywords were used “women,” “gender,” “children,” “minors,” “underaged, “pregnancy,” and “motherhood.” The authors of the respective countries also reviewed the full report to identify missing relevant text matter. For searching non-English documents, keywords were translated into the language of the concerned policy document. Non-English language texts were translated into English by the concerned author. Text was extracted into a Google Spreadsheet. Content analysis was performed on these texts/excerpts.

### Analysis

After thoroughly reading the text and data immersion, DJ and AG independently generated codes to indicate specific concerns for women, pregnant women/pregnancy, and women with children. Codes were also generated for the strategies discussed to address these concerns. The words, phrases, sentences, and paragraphs of text extracts were double coded. Coding categories were derived directly and inductively from the raw data using the constant comparative method. The coders checked the coding consistency across documents. An explicit or manifest coding approach was used to minimize the effects of subjective judgments on analysis [21]. Coded words and phrases were sought and the analysis did not go into the message behind the text. Frequently mentioned and similar codes were combined to generate themes. Coding discrepancies between DJ and AG were reflected upon and resolved by discussion. A repeat summative analysis by counting the occurrence of a particular theme in the reviewed policy documents was performed. This quantification was an attempt not to infer meaning but to explore usage. The quantitative information indicated the relative focus of the policies on specific themes across the areas of interest. Microsoft Excel was used for the analysis.

A thematic map was developed to integrate various codes and themes, understand how national-level drug policies are conceived, and further address the specific concerns for women, pregnancy, and motherhood.

An adapted version of the UNODC checklist for gender mainstreaming in projects/programs was used to assess the national-level policies/strategies/action plans adherence to international guidelines [22]. The checklist was developed in 2021, envisioning the UN requirement of a global strategy for promoting gender equality in any planned action, including legislation, policies, or programs. The original checklist has 14 items which rates adherence as “yes” or “no.” The items are classified into three groups: situation analysis (n = 5), plan description (n = 3), and plan management (n = 2). Situation analysis asks about the magnitude of drug problems among women, pregnant women, and women with children, and seeks to understand whether the differential impact of national strategies and international best practices are considered and how the policy aims to reach and empower women with drug misuse. Plan description includes the locus of care, provision of gender-sensitive care, and gender-responsive indicators. Plan management comprises logistics and financial support, and implementation measures. The checklist was adapted to include only 10 items. Three unrelated items were removed (i.e. staffing, resource mobilization, counterpart capacity) and two items were combined. The items were rated on a seven-point Likert scale (“strongly disagree” to “strongly agree”) because adherence should lie in a continuum. DJ made the initial adaptation and shared the checklist with AG, who reviewed and commented on the adaptation. The agreed version was shared with the research team for their comments and suggestions, and the checklist was finalized. The final checklist was shared with the research team for rating the adherence of their national policies to each of the items.

## Results

### Brief description of the studied documents

Twenty documents from nine countries were reviewed; Botswana, Croatia, Egypt, India, The Netherlands, North Macedonia, Poland, Portugal and Spain. Two national-level drug policy/program/action plan documents were retrieved from the following countries: Croatia, Portugal and Spain. The highest number (n = 5) of documents reviewed was from Poland. Three relevant drug policy documents were found from The Netherlands and one from North Macedonia. No drug policy-specific document was found for Botswana; however, a member of the research team reviewed the country's alcohol and health policies. Except for The Netherlands, all countries mention women, pregnancy, and motherhood-related concerns in a common drug policy document or national strategies. Only the Netherlands has a specific document directed towards pregnant women, a factsheet produced by the Dutch Association for Obstetrics and Gynaecology, “Vulnerable pregnant women and protection of the unborn child” which is part of a series about (domestic) violence, abuse, neglect, exploitation and other types of harm that may be inflicted onto someone in a power-imbalanced relationship [23].

Original publication of all these documents was in the last decade, except for “Counteracting drug addiction,” a drug demand reduction policy report from Poland, published in 2005 [24], and the Dutch policy, published in 1995 [25]. Five of the nine countries (Egypt, India, Croatia, Spain, and Poland) updated/ revised their drug policy in recent years [26–31]. North Macedonia brought out its latest drug policy in 2021 [32]. Two countries (Portugal and Botswana) have not updated their policies in the last ten years [33–35]. In most countries (n = 5), the Ministry of Health was responsible for publication of drug policy reports. However, different ministries in India, Poland, and Botswana have issued drug/alcohol policy documents, namely the Ministry of Social Justice and Empowerment, the Ministry of Finance/Trade, and the Ministry of Education. Spain has specific Ministerial involvement for drug policy measures and implementation, the Government Delegation for the National Drug Plan [36]. All nine countries intend to use the state budget to implement drug programs.

Table 1 provides further details.

**Table 1:** Description of national drug policy/programs/action plans

Country	Policy documents	Year of original publication	Updated	Ministry/department	Funding source
Botswana	1. National Alcohol Policy 2. National Health Policy 3. Southern African Development Community Health Protocol	2010 2011 1999	No	Ministry of Trade and Industry Ministry of Health	State
Croatia	1. National Strategy on Combating Drug Abuse in the Republic of Croatia for the Period 2012–2017 2. National Strategy of Actions in the Field of Addiction for the period until 2030	2012 2023	No No	Government of the Republic of Croatia, Office for Combating Drugs Abuse (inter-ministerial body) Government of the Republic of Croatia (budget), Ministry of Health, Croatian Public Health Institute (coordination and monitoring)	State budget State budget, EU funds, Lottery budget, local and regional municipalities
Egypt	1. Drug situation and policy	2014	No	Ministry of Health and population	–
India	1. Scheme of National Action Plan for Drug Demand Reduction 2. National Policy on Narcotic Drugs and Psychotropic Substances	2013 2012	Yes (2021) No	Ministry of Social Justice & Empowerment Ministry of Finance, Department of Revenue	State budget
The Netherlands	1. Dutch drug policy—continuity and change 2. National Prevention Agreement 3. Vulnerable pregnant women and protection of the unborn child	1995, 2009 2018 2020	No 2022 No	Ministry of Health; Ministry of Justice Ministry of Health Welfare and Sport Dutch Association for Obstetrics and Gynaecology	Budgets of the state, cantons and entities
North Macedonia	1. National Drugs Strategy of the Republic of North Macedonia 2021–2025 with Action Plan 2021–2023	2021	New Strategy	Ministry of Health	Ministry of Health, PHI,EMCDDA, UNODC,TAIEX EU program, Relevant institutions and/or donors
Poland	1. Act of 29 July 2005 on counteracting drug addiction 2. Regulation of the Council of Ministers of March 30, 2021 on the National Health Program for 2021–2025 3. Public Health Act of 11 September 2015 4. Regulation on educational activities in schools and educational system institutions to counteract drug addiction 5. Regulation of the Minister of Justice on treatment, rehabilitation and reintegration of addicted persons in district educational centres, correctional facilities and juvenile shelters	2005 2021 2015 2015 2022	2012, 2016–20 No 2016, 2017, 2019–2022 No No	Sejm of the Republic of Poland Sejm of the Republic of Poland Sejm of the Republic of Poland Ministry of Education Ministry of Justice	State Regional, Municipal
Portugal	1. National Plan for the reduction of addictive behaviours and dependencies 2013–2020) 2. Action Plan for Reduction of Addictive behaviours and dependencies 2013–2016	2013 2013	No No	General Directorate for Intervention on Addictive Behaviours and Dependencies (SICAD) General Directorate for Intervention on Addictive Behaviours and Dependencies (SICAD)	State budget State budget
Spain	National Strategy on Addictions 2017–2024	2017	Yes, 2021	Ministry of Health and Government Delegation for the National Drug Plan	State Regional, Municipal

### Content analysis of the national policy/programs/strategies

We describe the results of the conventional content analysis as per “women,” “pregnancy,” and “children” sensitive/responsive texts in the reports.

- a. Women-sensitive policy

We identified 19 unique *codes* from the content analysis. The recurrent and similar *codes* were combined to generate 11 major themes. The excerpts from the policy documents were quoted and presented in Table 2.

- 1. Special concerns in women

**Table 2:** Themes, Codes and Excerpts for “Women-sensitive Policy”

Excerpts	Codes	Themes
Women—sensitive policy		
“In this context, it is important to integrate gender violence as one more element in the approach to addictions. In the case of a substance use disorder, the rates of gender violence are three times those in the general population. It is necessary to work on intervention with both men and women [34]”	Gender violence	Special concerns in women
“…..consumption little visible, greater stigma and blame and less support and understanding, more judgment reinforced by ideas of motherhood and the impact of children” “It is necessary to support the organisations of treated addicts and shift more focus to programmes aimed at female population of addicts (specially adapted programmes of field work and informing on different risks related to drug use, including prostitution and threat to the infant during the pregnancy of a drug addicted mother) [31]”	Stigma, Shame, Blame	
“…consider family responsibilities (as these fall overwhelmingly on women) as a possible barrier to accessing the care network and in the process of rehabilitation and social reintegration [30]”	Gender role	
“Protection of vulnerable populations such as youth and women—guidance provided under the alcohol policy is based on the fundamental principle that it is up to the Government to protect its citizens from alcohol-related harm, particularly harm to women and youth who are more vulnerable to developing alcohol related conditions [35]”	Vulnerability	
“Specialized training for those who work with vulnerable groups, such as patients with psychiatric co-morbidities, children and women, including pregnant women [27]”		
“A National Survey of Drug abuse was conducted in 2001. It had three major components (i) National Household Survey, (ii) Rapid Assessment Survey and (iii) Drug Abuse Monitoring System, which analyzed the profile of treatment seekers. There were sub-studies on drug abuse among rural population, prison population, women, and in border areas. The survey and studies indicated that commercial sex workers, transportation workers, and street children are at greater risk of drug addiction than the general population [27]”	Conduct surveys	Needs Assessment
“…to understand and integrate the needs of women with drug misuse. Organic Law 3/2007, of March 22, for the equality of women and men (art. 20) states that public administrations must integrate a gender perspective into their studies and statistics as an analytical framework and develop tools which allow for a better understanding of the impact of gender” “In the context of the Spanish National Drug Plan and National Addictions Strategy this analytical framework should be used to enable making visible, analyzing and addressing the different presentation and impact of addictions in women and incorporate the needs and reality of women in all actions and strategic interventions [37]”	Gender-responsive analytical framework	
“Pilot early detection and brief intervention programs for minors, women, and sexual and reproductive care services, family planning, pre-pregnancy visits, and obstetrics, as well as sexually transmitted infection units [31]”	Early identification and treatment	Prevention
“Promote programs focused on the needs of women (for example, dependence on prescription drugs), analyzing all aspects related to consumption and developing alternatives for non-drug treatment [31]”	Gender-sensitive treatment	Treatment
“Establishing and assisting de-addiction centers in closed settings such as Prisons and Juvenile Homes and for special groups such as women and children in need for care and protection, etc. through State Government [27]”	Custodial settings	
“Through active employment measures of the Ministry of Labor and Social Policy and the Employment Agency of Republic of North Macedonia (EARNM), employment opportunities are being offered to members of marginalized communities through stimulations and benefits for employers, and for persons seeking job who get opportunities to attend training qualifications [32]”	Creating social and economic opportunities	Social reintegration
“The United Nations Convention against Transnational Organized Crime and the Protocol to Prevent, Suppress and Punish Trafficking in Persons, Especially Women and Children, supplementing the United Nations Convention against Transnational Organized Crime was ratified by Egypt and the instrument of ratification was deposited on 5 March 2004 [26]”	Interrupting drug trafficking	Supply reduction
“Specialized training for those who work with vulnerable groups, such as patients with psychiatric co-morbidities, children and women, including pregnant women [27]” “Carrying out continuous training of the medical staff working on programs for implementation of adapted programs for dependency treatment of children and women. As well as carrying out continuous training among CSOs for the implementation of new programs for women and transgender persons [32]” “Encourage the creation of the capacity in existing and new therapeutic communities for minors, addicted women and drug addicts with dual diagnoses (comorbidity), and carry out targeted educational trainings for all employees in homes for addicts and therapeutic communities, including professional staff, rehabilitated addicts and volunteers [28]”	Capacity building	Training
“………National Backward Classes Finance and other Development Corporations of the Ministry of Social Justice and Empowerment. In addition, vocational training and livelihood programmes would be carried out in collaboration with the Ministry of Women and Child Development, Ministry of Skill Development and Entrepreneurship and its affiliated institutes and State Governments [27]”	Inter-miniterial, inter-sectoral collaboration	Collaboration and Coordination
“Guarantee quality integrated care” which includes the coordination of resources for women who suffer gender-based violence and their children [31]”	Integrated care	
“All indicators should be collected and presented broken down by age and gender, where data is available [38]” “Effectively incorporate the gender perspective as a tool for analyzing reality, in all programs, investigations and intervention and prevention.” [31]	Gender-sensitive indicators	Monitoring and evaluation
“Addressing gender sensitive and responsive issues, including the equal involvement of men and women in decision-making, eliminating obstacles (barriers) to services utilization, and the prevention of gender-based violence.” [39]	Gender-sensitive policy	Policy
Expanding the Gender budgeting and planning approaches that are responsive to women’s needs in various sectors: adopting policies, planning approaches and budgets responsive to women’s needs are effective methods to bring about social change; through the allocation of resources to implement policies and programs entitled to reduce the gender disparities and eliminate obstacles depriving women of accessing the public services.” [41]	–	Resource allocation

Several policy documents discussed the need to recognize and address special issues in women with drug use. *Vulnerability*, *shame*, *blame*, *stigma*, *gender role*, and *gender violence* were specifically mentioned.

- 2. Needs assessment

Needs assessment refers to a practice for estimating the nature and magnitude of a health or social problem in a community/state/country where there is the intent to ameliorate or otherwise respond to that problem. The drug policy documents we reviewed described the need to *conduct surveys* among women and at-risk populations, not only to understand the problem but also to design acceptable and effective strategies to address it. Spain’s policy, in addition to the importance of conducting drug surveys, discusses a *gender-responsive analytical framework* to understand and integrate the needs of women with drug misuse.

- 3. Prevention

The prevention strategies discussed in the documents included both universal actions, such as raising awareness in the general population, and the need for selective and indicated prevention programs and interventions targeting women.

- 4. Treatment

Drug policy measures from various countries emphasize the role of *gender-sensitive treatment*. India’s National Action Plan for Drug Demand Reduction (2018–2025) talks about the provision of treatment in closed custodial settings and combined women with other vulnerable populations [26].

- 5. Social reintegration

Policy measures are needed to develop and promote interventions and strategies to address housing, education, vocational training and employment. These interventions can be grouped as strategies for social reintegration. North Macedonia’s national health policy talks about providing social and economic opportunities [32].

- 6. Supply reduction

Supply reduction means using strategies to disrupt the production and supply of illicit drugs. *Interrupting drug trafficking* along the shipping, air, and road transport routes is one supply reduction measure. The relationship between women and the drug trade is complex. Women’s participation in the drug supply chain can be attributed to vulnerability and oppression, where they are forced to be involved out of fear or exploitation. Another narrative explains the involvement in the drug trade as their own decision. Women may be involved in trafficking to sustain their drug consumption or be victims of in-person trafficking [4].

- 7. Training

The national policies recognized the need for workforce training and capacity building in gender-responsive prevention and treatment. The training need extends to non professional voluntary workers and peer groups.

- 8. Collaboration and coordination

Several national policies mentioned the need for *inter-ministerial*, *inter-sectoral*, and *international collaborations* to achieve the objectives of the policy documents. The Spanish National Strategy on Addictions states as a strategic objective, “Guarantee quality *integrated care*” which includes the coordination of resources for women who suffer gender-based violence and their children [31].

- 9. Monitoring and evaluation

Periodic surveillance is required to inform policymakers about existing drug policies’ effectiveness and limitations. Only a few of the countries’ policies reiterated the importance of *gender-sensitive indicators* for monitoring the implementation and outcome of the policy measures.

- 10. Policy

Policies are *guiding principles* chosen by a government and dictate the strategies and action plans to address a particular issue, e.g. control and regulation of psychoactive drugs.

- 11. Resource allocation

Resource allocation refers to assigning available resources to various uses.

- b. “Pregnant women” sensitive policy

We discovered five major themes from nine different *codes* from the content analysis of the policy text concerning pregnant women. Four of the five themes have already been discussed while discussing women-sensitive policy. The only new theme that emerged in this section is the custody of children of women with drug misuse. Please see Table 3 for the excerpts from the policy documents, codes and themes.

- 1. Prevention

**Table 3:** Themes, codes and excerpts for “pregnant women & children-sensitive policy”

Excerpts	Codes	Themes
Pregnant women		
“Sensitize and inform pregnant women and their partners about the impact of their addictive behaviors on fetal development…” [33]	Education and awareness generation	Prevention
“…initiating, supporting and conducting activities in the field of prevention of disorders that result from prenatal exposure to alcohol, narcotic substances, psychotropic drugs, substitutes or new psychoactive substances, as well as initiating and supporting activities in the field of helping people with fetal alcohol spectrum disorders (FASD) and their families or carers [39]”	Preventing exposure to psychoactive substance during pregnancy	
“Early detection and treatment in the sexual and reproductive care services, family planning, pre-pregnancy visits, and obstetrics, as well as sexually transmitted infection units [31]”	Screening	
“Mobilize the teams that, at the level of primary health care, develop maternal and child health responses, through a process of training and technical framework by the services specialized in CAD.” [31]	Integrated care	Treatment
“Ensure priority access to specific care for pregnant women with drug and alcohol misuse and their partners, according to their problems and risk levels in the course of pregnancy… [31]”	Gender-sensitive treatment	
“Unborn children can be put under supervision by a social worker throughout pregnancy. Child protection can then give instructions such as assisting with obstetric check-ups and other forms of care. (..). In the case of suicidality and addiction to hard drugs, forced admission to a care facility is possible based on the BOPZ Act. In the event of incapacity in making decisions, mentorship or guardianship can be requested from the court.” [23]	Child care	Child custody issues
“Specialized training for those who work with vulnerable groups, such as patients with psychiatric co-morbidities, children and women, including pregnant women.” [27]	Training	Training
“Strengthening the articulation between services, in order to allow an integrated intervention at different levels (health promotion prevention, treatment, reinsertion, and harm reduction), in multiple contexts at the stage of pregnancy and neonatal period, englobing the family contexts community, prisional settings [31]”	Multilevel collaboration	Collaboration and Coordination
Child-sensitive policies (Additional themes)		
“The State is responsible for ensuring the protection of children and adolescents through legal regulations when other measures are insufficient to provide effective protection. Legal measures must find adequate responses to new challenges [31]”	Reduce supply/access to drugs	Legislation
“Study and identify factors and processes that increase the risk of developing addictive behaviors without substances [33]”	Preventive research	Research
“Harm reduction should also include protection of the health and safety of minors and other family members in settings affected by drug use. There is evidence that people’s substance misuse can have an impact on the lives of the people around them, especially the family.” [31]	Minimize harm to others	Harm reduction

Universal prevention, such as *education/awareness generation* of pregnant women and their partners regarding the harmful effects of drugs on fetal development. Some policy texts broadened the scope of prevention by incorporating *prevention of exposure to psychoactive substances during pregnancy*, further preventing the fetal neurobehavioral and developmental consequences. As mentioned earlier, Spanish policy promotes *screening, early identification, and brief interventions* for alcohol in “…sexual and reproductive care services, family planning, pre-pregnancy visits, and obstetrics, as well as sexually transmitted infection units” [31].

- 2. Treatment

This was largely discussed in the context of gender-sensitive treatment, in the context of women and pregnant women together, or with an exclusive focus on pregnant women. The Portuguese policy also aspires to deliver a seamless and integrated model at all levels of care.

- 3. Custody issues

*Child custody* refers to a child's care, control, and maintenance through a legal mandate. The court aims to look after a child's best interest in deciding custody rights. Penalties for substance use in pregnancy can include loss of custody, compulsory treatment*,* and fines. [23, 40]. Fear of loss of custody can lead to some women avoiding seeking treatment [20].

- 4. Training

Training of human resources was discussed with the need for specialized gender, and youth sensitive training on the prevention and treatment of drug misuse. For example, the Indian drug demand reduction policy stated, “Specialized training for those who work with vulnerable groups, such as patients with psychiatric co-morbidities, children and women, including pregnant women.” [27].

- 5. Collaboration and coordination

A few national drug policies expressed the need for coordination between various levels of service delivery systems: prevention, treatment, and harm reduction and also collaboration between services for women, pregnant women, and their children [27, 32, 33].

- c. Children-sensitive policy

The content analysis of policy documents that pertained to children revealed 20 unique *codes* and 10 major themes. Most themes (n = 7) were similar to those captured and already described under the women/gender-sensitive policy matters, such as needs assessment, prevention, treatment, supply reduction, training, collaboration and coordination, and policy. Additional themes discovered were legislation (to protect children), resource allocation, research, and harm reduction. Below we discussed only those themes exclusive to “children”/“children with a parent who uses drugs.” The excerpts supporting the codes and additional themes were presented in Table 3.

- 1. Legislation

Legislation signifies creating and implementing laws to prevent the production/manufacture/cultivating, possessing, selling, purchasing, transporting, storing, and/or consume any psychoactive substances. It aims to *reduce the supply/access to drugs* to those who use drugs.

- 2. Research

Research is an integral component of drug policy. Preventive intervention research is important in this particular context of drug policies on children. Prevention may target the at-risk population, which is known as selective prevention. Portuguese policy alludes to the commitment to conducting *risk-factor research*.

- 3. Harm reduction

Harm reduction refers to policies, programs, and practices that minimize drug use’s negative health, social and legal impacts. Harm reduction was cited in the Spanish national strategy, not for children, but for adults in order to protect the children in the family i.e., to minimize *harm to others*.

### Summative quantitative analysis

**Fig. 1:**
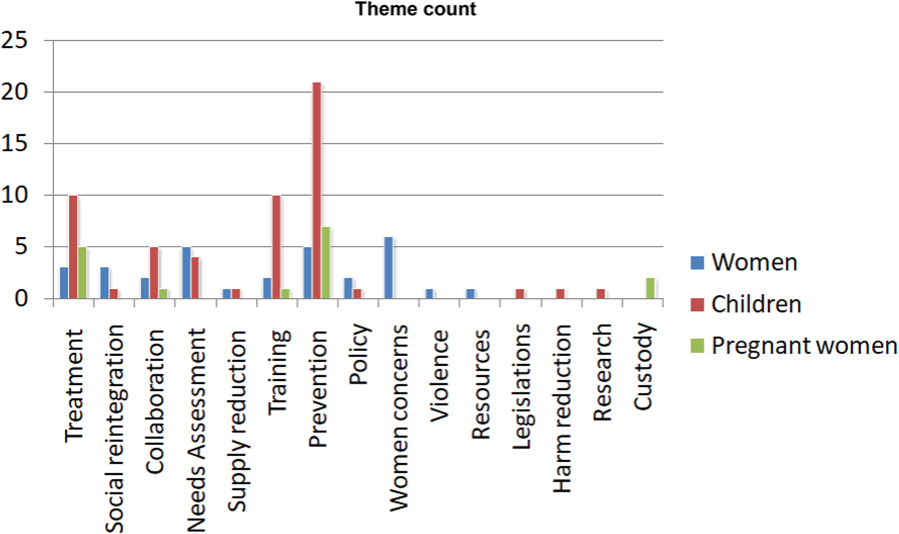
Summative analysis of the category-wise theme frequency (occurrence of a particular theme in the policy documents)

Figure 1 depicts the number of times a particular theme occurred during the content analysis. Because the themes were different for three categories (e.g., women, pregnant women, children) and we wanted to check the relative frequency of occurrence of a particular theme in a specific category, we presented the “theme count” category-wise.

For women-specific content analysis, special issues/concerns in women with drug misuse, need assessment, and prevention were the three most frequent themes; for the children-specific policies, prevention, training, and treatment comprised the three most occurring themes. For pregnant women's sensitive policy texts, prevention, treatment, and child custody were the highest-occurring themes.

### Thematic map

**Fig. 2:**
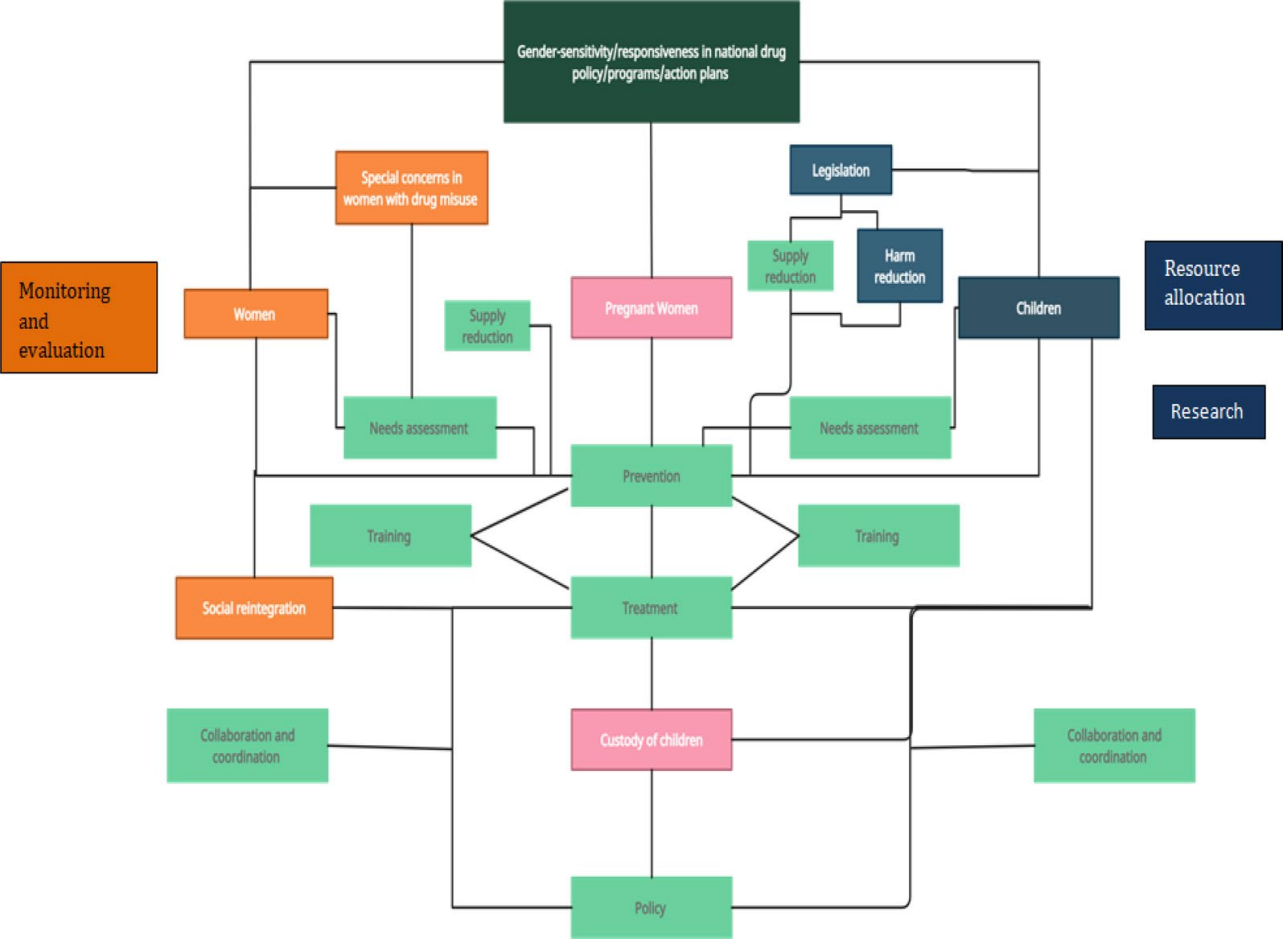
Thematic map for visualizing inter-relationships between concepts and among main themes and subthemes. Green colored boxes are themes common to women, pregnant women, and children. Orange colored boxes are themes unique to women. Pink colored boxes contain unique theme for pregnant women. Blue colored boxes are children-specific themes

Figure 2 illustrates the thematic map to visualize the cross-connections between common and unique themes (specific to the target population) discovered during the content analysis. All policies/programs are envisioned to prevent and treat drug misuse among women, pregnant women, and children. They emphasized the importance of needs assessment to understand the magnitude and patterns of the problem. The prevention can be universal (that is desirable for everybody in the eligible population, e.g., raising awareness and education at the population level), selective (identifying and addressing concerns of the vulnerable population), and indicated (early detection of risk factors and condition, and providing intervention to prevent future development of SUD). However, some policies reported the need for supply reduction and legislation to protect children and women (and the unborn children of pregnant women) as preventive measures. The policies talked about training and capacity building of human resources to deliver gender-sensitive care. Special psychosocial concerns in women with drug misuse, such as the stigma, blame, gender-based violence, and typical gender-role, must be examined and addressed in a gender-sensitive prevention and treatment program. Following treatment, national drug policies envisage providing socio-economic opportunities for the social reintegration of women with drug misuse. Drug policies deliberated on the child custody issues for pregnant women with drug misuse and linked this with the treatment. Implementing the policy measures will require inter-ministerial and international collaboration and coordination.

The performance of policies must be periodically monitored by gender-sensitive indicators and adapted to the changing needs and demands.

### Adherence of the national policy/programs/strategies to the adapted checklist for gender-sensitive policy

Each author rated their own country’s policy reports. According to the ratings of the country’s gender-sensitive policy, there is limited adherence to international guidelines and lessons learned on gender equality and women’s empowerment, and engendering results chain which ensures outcome, output, and activities are in sync with the specific need in women and for pregnancy, and motherhood. Five and six out of nine members responded with different grades of disagreements (regarding their country’s policy adherence) on the likert scale for these two items, respectively.

**Fig. 3:**
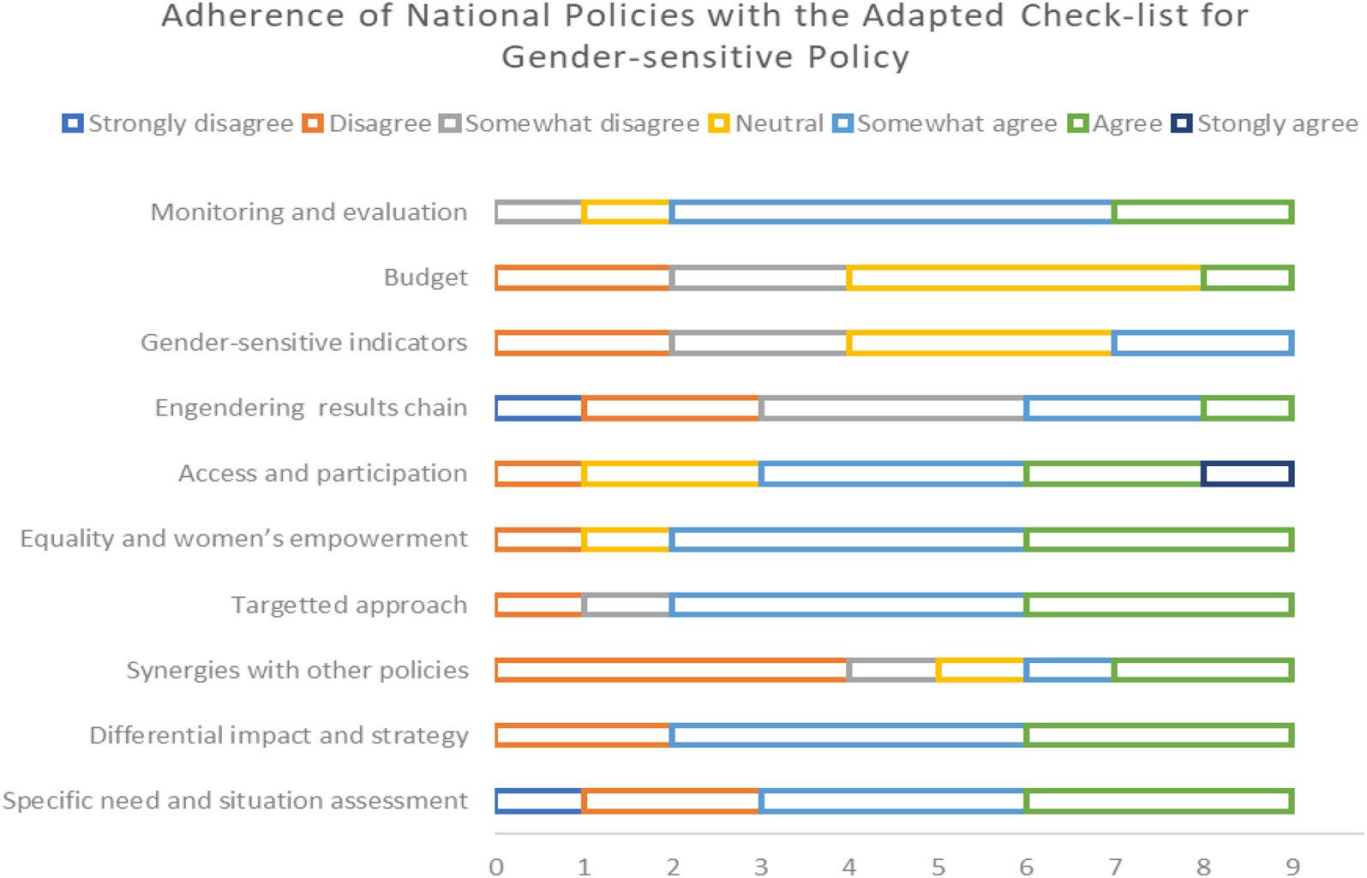
Adherence of national policies with the adapted check-list for gender-sensitive policy

Very little agreement was reached regarding the provision of gender-responsive indicators (agreement = 2/9) and targets and adequate and sustainable financial resources to implement the components of the policy (agreement = 1/9). The adherence is relatively better for the background situation and context analysis (agreement = 6/9), consideration of differential impact and strategies (agreement = 7/9), targeted approach to reach out to this special population (agreement = 7/9), envisioning equality and women empowerment (agreement = 7/9), improving access and participation in interventions (agreement = 6/9), and monitoring and evaluation of the implementation of the policy measures (agreement = 7/9). The agreement includes those who at least “somewhat agreed” to the checklist items. See Fig. 3 for finer details.

## Discussion

Gender differences in drug use patterns, characteristics, and intervention needs represent an important policy issue addressed to various extents in different countries [42]. Our first objective was to systematically explore national-level drug policies/strategies/action plans’ sensitivity and responsiveness to women, pregnancy, and motherhood.

The content analysis showed common and unique themes for all three included categories. For women, pregnant women, and children among the most frequently mentioned themes were prevention and treatment. This can be seen as a continuum of drug demand reduction responses relevant for people with substance-use disorders and their families that most of the included countries recognized as relevant [43]. Special concerns and needs assessment were among the most frequent themes for women. Women who use drugs have high rates of mental health problems and histories of childhood abuse, and greater vulnerability to drug-related harms [40, 44]. Our analysis shows that these issues have been addressed in the context of gender-sensitive drug policy responses in some (but not all) countries. Needs assessment was recognized as an important factor in providing adequate care. This aligns with research showing that combining integrated services based on the needs assessment can result in positive outcomes for individuals, families, and society [12, 43, 45].

The importance of child custody for children of mothers with drug-related disorders was considered a unique issue and the most frequent theme for pregnant women. Child custody has a long-term effect on children throughout their adulthood. Faherty et al. [14] emphasize that penalties for substance use in pregnancy can include loss of custody, which can have repercussions on children’s development and produce additional expenses for society [14]. Pregnant women with substance use disorders should get the care and support they need, not only in the context of treatment but also through supporting their motherhood [6, 12, 15, 16]. However, this theme also reflects the gendered differences in the perception of substance use and its conflict with the traditional gender role, where mothers are expected to care for and raise children, not fathers [46].

Among the most frequent themes for the children-specific category was training. Most countries recognize the importance of trained professionals when delivering interventions, which aligns with the guidelines for delivering effective interventions [47], and is supported and recognized by relevant international bodies [7]. While the themes found generally align with research evidence, it is unclear how much this has influenced the country’s policies.

The policy documents used stigmatizing language to depict persons who use drugs. Words and phrases such as “addicts,” “Addicted mother,” “drug users,” and “addicted women” were used in various policy documents. Although language did not typically fall under the purview of our work, we decided to code and generate additional themes because of the harmful effect of stigmatizing language in propagating public stigma. The use of inappropriate language in the policy documents might contribute to the public stigma against persons who use drugs. We recommend using neutral and person-centered language in all public and scientific documents on substance use [48]. This is more significant in women who use drugs because of the gendered perception of drug use.

Our second objective was to examine the adherence of drug policies/strategies/action plans with the international guidelines for gender sensitivity in drug policy. Findings showed that areas that need additional focus in national policies include budget, gender-sensitive indicators, engendering results chain, and synergies with other policies. It seems that a minority of the analyzed policies ensure adequate financial resources specifically for the activities for women, pregnancy, and motherhood. Likewise, gender-sensitive indicators and outcomes, outputs, and activities are not designed to a large extent to meet the different needs and priorities of included categories. Finally, the lack of synergies with other policies was recognized as an important area that can be improved by accepting the coherent approach to substance-use policies [49].

Although our analysis was limited to nine countries, our results showed some general drug policy lacunae that might apply to other countries. Gendered perception of drug use, a wide policy-practice gap resulting from inadequate resources and limited availability of gender-sensitive data, lack of synergy with other health policies, and potentially harmful and stigmatizing language use came up as significant concerns which need to be addressed.

### Limitations

This study has several limitations that need to be acknowledged. Firstly, included countries came mostly from Europe and are high- or middle-income countries, which decrease the generalizability of the findings. There are differences among included countries regarding the infrastructure and responses to drug use. In addition, the chosen topic does not present an equal political priority in all included countries. In terms of policy, there is a lack of documents and national guidelines dedicated specifically to women using drugs, and also no civil society official documents were considered which means that we might be lacking a view that could complement state policies (or the lacking of gender specific approaches). Findings related to our first objective are based on content analysis, which has a component of subjectivity. Furthermore, group members came from different backgrounds and were not policy analysts by profession. Our second objective was to examine the adherence of the analyzed documents with the international guidelines for gender sensitivity in drug policy. This approach also has several limitations. First of all, the adapted Checklist focuses on women, pregnancy, and motherhood, but the situation might not necessarily be the same for the three issues. For instance, in some policy documents, there is almost no reference to pregnancy, so it is possible that an assessment was not made for all three target groups/terms in the included countries. Another area for improvement is that in the adapted Checklist, there was no option to not respond to a question, even though some questions perhaps required more information and knowledge than our group members had. Finally, each member analyzed their national policy, which might have resulted in bias and a degree of subjective interpretation. Adding a second rater could have improved the robustness; however, because of the unfamiliarity with the language of the documents and country context/situation, we had to rely on a single rater.

## Conclusion

In sum, our research evinced the components in the national drug policies/documents that address special concerns in women, pregnancy, and motherhood. We identified areas that are either neglected or inadequately addressed and require further strengthening. We believe our analysis would stimulate discussion, critical review of national policies, and inclusion of gender sensitivity as a conscious and deliberate decision to meet the goals of United Nations SDG 2030 (Goal 5: Achieve gender equality and empower all women and girls).

## Data Availability

The URLs of the documents analyzed for the report are added to the references. Coding, thematic analysis, and adherence rating data supporting this study’s findings are available from the corresponding author, [DJ], upon reasonable request.

## Abbreviations

CSO: Civil Society Organisations
EMCDDA: European Monitoring Centre for Drugs and Drug Addiction
EU: European Union
FASD: Fetal alcohol spectrum disorders
Inter-GLAM: Intercontinental Perspectives on Global Addictions and Drug Markets
JUST-2019-AG-DRUGS: European Union’s DG Justice Programme “Drugs Policy Initiatives—Supporting initiatives in the field of drugs policy”
Misuse: Harmful/hazardous use, use disorder, dependence
PHI: Public Health Institute
SICAD: General Directorate for Intervention on Addictive Behaviours and Dependencies
EARNM: Employment Agency of Republic of North Macedonia
SUD: Substance Use Disorders
TAIEX: Technical Assistance Instrument and Information Exchange Instrument of the European Commission
UN: United Nations
UNODC: United Nations Office on Drugs and Crime
WHO: World Health Organisation
